# Heme Biosynthesis Factors and 5-ALA Induced Fluorescence: Analysis of mRNA and Protein Expression in Fluorescing and Non-fluorescing Gliomas

**DOI:** 10.3389/fmed.2022.907442

**Published:** 2022-05-18

**Authors:** Mario Mischkulnig, Thomas Roetzer-Pejrimovsky, Daniela Lötsch-Gojo, Nina Kastner, Katharina Bruckner, Romana Prihoda, Alexandra Lang, Mauricio Martinez-Moreno, Julia Furtner, Anna Berghoff, Adelheid Woehrer, Walter Berger, Georg Widhalm, Barbara Kiesel

**Affiliations:** ^1^Department of Neurosurgery, Medical University of Vienna, Vienna, Austria; ^2^Comprehensive Cancer Center–Central Nervous System Tumours Unit, Medical University of Vienna, Vienna, Austria; ^3^Division of Neuropathology and Neurochemistry, Department of Neurology, Medical University of Vienna, Vienna, Austria; ^4^Department of Neurosurgery, University Hospital of St. Poelten, Karl Landsteiner University of Health Sciences, St. Poelten, Austria; ^5^Department of Neurosurgery, Vivantes Klinikum Neukölln, Berlin, Germany; ^6^Department of Radiology and Nuclear Medicine, Medical University of Vienna, Vienna, Austria; ^7^Clinical Division of Oncology, Department of Medicine I, Medical University of Vienna, Vienna, Austria; ^8^Institute of Cancer Research, Medical University of Vienna, Vienna, Austria

**Keywords:** 5-ALA, fluorescence, gliomas, heme biosynthesis, mRNA expression, protein expression

## Abstract

**Objective:**

The intraoperative visualization of adult-type diffuse gliomas with 5-aminolevulinic acid (5-ALA) induced fluorescence is widely used in the neurosurgical field. While visible 5-ALA induced fluorescence is found in the majority of high-grade gliomas, most low-grade gliomas lack visible fluorescence during surgery. Recently, the heme biosynthesis pathway was identified as crucial influencing factor for presence of visible fluorescence since it metabolizes 5-ALA to fluorescing Protoporphyrin IX (PpIX). However, the exact alterations within the heme biosynthesis pathway resulting in visible 5-ALA induced fluorescence in gliomas are still unclear. The aim of the present study was thus to compare the mRNA and protein expression of promising intramitochondrial heme biosynthesis enzymes/transporters in glioma tissue samples of different fluorescence behavior.

**Methods:**

A total of 19 strongly fluorescing and 21 non-fluorescing tissue samples from neurosurgical adult-type diffuse gliomas (WHO grades II-IV) were included in the current analysis. In these samples, we investigated the mRNA expression by quantitative real time PCR and protein expression using immunohistochemistry of the intramitochondrial heme biosynthesis enzymes Coproporphyrinogen Oxidase (CPOX), Protoporphyrinogen Oxidase (PPOX), Ferrochelatase (FECH), and the transporter ATP-binding Cassette Subfamily B Member 2 (ABCG2).

**Results:**

Regarding mRNA expression analysis, we found a significantly decreased ABCG2 expression in fluorescing specimens compared to non-fluorescing samples (*p* = 0.001), whereas no difference in CPOX, PPOX and FECH was present. With respect to protein expression, significantly higher levels of CPOX (*p* = 0.005), PPOX (*p* < 0.01) and FECH (*p* = 0.003) were detected in fluorescing samples. Similar to mRNA expression analysis, the protein expression of ABCG2 (*p* = 0.001) was significantly lower in fluorescing samples.

**Conclusion:**

Distinct alterations of the analyzed heme biosynthesis factors were found primarily on protein level. Our data indicate that heme biosynthesis pathway activity in general is enhanced in fluorescing gliomas with upregulation of PpIX generating enzymes and decreased ABCG2 mediated PpIX efflux outweighing the also increased further metabolization of PpIX to heme. Intramitochondrial heme biosynthesis factors thus constitute promising pharmacological targets to optimize intraoperative 5-ALA fluorescence visualization of usually non-fluorescing tumors such as low-grade gliomas.

## Introduction

Adult-type diffuse gliomas are the most common primary malignant tumors of the central nervous system (CNS) (1). According to the World Health Organization (WHO) classification, these gliomas are subdivided into three different grades (WHO grades II, III, and IV) (2). Generally, the initial treatment consists of neurosurgical resection of gliomas whenever this is safely possible (2, 3). With regard to surgery, the extent of resection was identified as major prognostic factor in gliomas (4, 5). Thus, the goal of surgery represents maximal safe tumor resection with preservation of neurological function (6, 7). However, incomplete resection of gliomas is observed in a large portion of patients in routine clinical practice mainly due to insufficient intraoperative visualization of (residual) tumor tissue leading to worse patient outcome (8).

Fluorescence-guided resection using 5-aminolevulinic acid (5-ALA) has become a widely applied technique to improve intraoperative tumor visualization and thus the extent of resection in brain tumors (8–10). This innovative technique is especially useful to visualize high-grade gliomas (HGG; WHO grades III and IV) during surgery due to the very high rate of strong fluorescence (10, 11). In this sense, visible 5-ALA induced fluorescence is found in almost all cases of WHO grade IV gliomas and approximately 70–80% of WHO grade III gliomas (10, 11). Aside from HGG, 5-ALA was also investigated in other tumors such as diffusely infiltrating low-grade gliomas (LGG; WHO grade II) (11–13). In contrast to HGG, visible 5-ALA induced fluorescence was found in previous studies in only 10-20% of LGG during surgery (11, 14). Therefore, 5-ALA induced fluorescence is generally not useful to improve the extent of resection in pure LGG (15, 16). The understanding of the underlying alterations resulting in visible fluorescence in different gliomas is not fully clarified so far (17). Nevertheless, visualization of tumor tissue would be of major importance, especially during surgery of LGG, due to the only slight macroscopic differences of tumor tissue compared to normal brain parenchyma (18).

**FIGURE 1:**
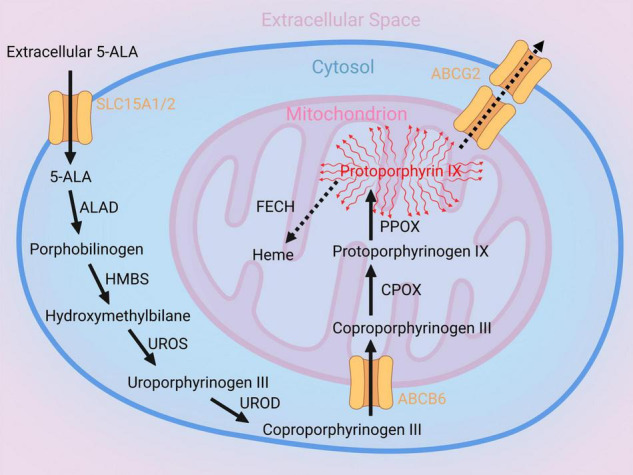
Illustration of the heme biosynthesis pathway catalyzing 5-aminolevulinic acid (5-ALA) to fluorescent Protoporphyrin IX (PpIX). After metabolization of 5-ALA within the intracellular space, the ATP-binding cassette super-family B member 6 (ABCB6), a mitochondrial protein, transports Coproporphyrinogen III from the intracellular space into the mitochondrion. Subsequently, Coproporphyrinogen III is decarboxylated by the Coproporphyrinogen oxidase (CPOX) and generates Protoporphyrinogen IX. In the next step, Protoporphyrinogen oxidase (PPOX) catalyzes Protoporphyrinogen IX to fluorescent PpIX. This fluorescent metabolite is on the one hand transported outside the cell by ATP-binding transporter Cassette Subfamily G member 2 (ABCG2) and on the other hand enzymatically modified to Heme by Ferrochelatase (FECH).

5-Aminolevulinic acid is a metabolic precursor of heme and is not fluorescent itself (9, 19). The heme biosynthesis pathway consists of different enzymatic steps catalyzing 5-ALA to fluorescing Protoporphyrin IX (PpIX) (see Figure 1; 19). One of these important enzymes is Coproporphyrinogen Oxidase (CPOX), which generates Protoporphyrinogen IX via oxidative decarboxylation (19). The next step consists of oxidation of Protoporphyrinogen IX mediated by Protoporphyrinogen Oxidase (PPOX) resulting in fluorescing PpIX (19). Subsequently, PpIX can either be transported outside the cell by ATP-binding transporter cassette subfamily G member 2 (ABCG2) or enzymatically modified to heme by Ferrochelatase (FECH) (19). While, the general metabolic heme biosynthesis pathway catalyzing 5-ALA to fluorescing PpIX is well-described, the exact mechanisms resulting in visible 5-ALA induced fluorescence in different gliomas is not clearly understood (20, 21). Recent studies have suggested effects of the intramitochondrial heme metabolizing enzymes CPOX and FECH on 5-ALA fluorescence behavior (21, 22). Identification of the relevant alterations in heme biosynthesis pathway regulation responsible for visible 5-ALA induced fluorescence in gliomas might allow modifications in heme biosynthesis activity to improve intraoperative visualization of usually non-fluorescing tumor tissues such as LGG in the future (22).

The aim of our current study was a comprehensive investigation of intramitochondrial heme biosynthesis factors in a series of gliomas WHO grades II, III, and IV reflecting the typical association of fluorescence intensity with WHO grade. To this end, we compared the mRNA as well as protein expression levels of CPOX, PPOX, FECH and ABCG2 in glioma tissue samples with presence of strong 5-ALA induced fluorescence during surgery to non-fluorescing glioma samples.

## Materials and Methods

### Study Cohort and Selection of Fluorescing/Non-fluorescing Glioma Samples

At our department, we established a 5-ALA databank including data of patients with intraoperative fluorescence application and information on available tissue samples as well as corresponding clinical, histopathological, and radiological data. In order to optimally investigate differences in the underlying heme biosynthesis pathway, we defined two different groups of approximately 20 glioma (WHO grades II, III, and IV) with 5-ALA administration with the largest difference of fluorescence behavior for the current study: (1) *fluorescing group* includes only tumors with strong 5-ALA induced fluorescence and (2) *non-fluorescing group* consists of gliomas with no visible fluorescence. For this purpose, we screened our 5-ALA databank for patients with a histopathologically confirmed and newly diagnosed adult-type diffuse glioma (WHO grades II, III, and IV) with neurosurgical resection between 2012 and 2020 and intraoperative use of 5-ALA induced fluorescence. Subsequently, we selected representative snap frozen samples (stored at −80°C) from gliomas with strong 5-ALA induced fluorescence as maximum fluorescence level as well as tumors with no visible 5-ALA induced fluorescence.

In the present study, we only included samples from adult patients aged 18 years or older at the time of surgery without previous treatments. This study was carried out in accordance with the Declaration of Helsinki and approved by the local ethics committee of the Medical University Vienna (*EK 419/2008*, amendment), all patients gave written informed consent prior to inclusion.

### Neurosurgical Tissue Collection and Histopathological Tumor Diagnosis

All included patients received a standard dose of 5-ALA (20 mg/kg body weight) approximately 3 h prior to anesthesia (8, 10). During tumor resection with navigational guidance, the neurosurgeon repetitively switched to violet-blue excitation light to determine the maximum intraoperative fluorescence status of each tumor (strong, vague, or no visible fluorescence) and corresponding tissue samples were collected as described previously (13). In order to allow collection of representative tumor tissue in gliomas with non-significant contrast-enhancement (none, patchy/faint or focal), tissue sampling from a potential metabolic hotspot detected by positron emission tomography (PET) using ^11^C-methionine (MET) or ^18^F-fluoro-ethyl-l-tyrosine (FET) was generally conducted (23–25). The collected tissue samples were divided into two parts: (1) first part was obtained for histopathological and immunohistochemical analyses and (2) the second part was immediately snap frozen and stored at −80°Celsius for further mRNA analyses. In all patients, histopathological tumor diagnosis was established or reclassified according to the current WHO classification of 2016 including assessment of the isocitratdehydrogenase (IDH) mutational status and 1p19q-codeletion (2, 26).

### Histopathological Analyses and Immunohistochemistry for Protein Expression

Initially, the first part of the collected tissue samples was formalin-fixed, paraffin-embedded and processed for histopathological and immunohistochemical analyses. In the first step, all samples were screened for the presence of vital tumor tissue as well as absence of extensive necrosis based on histopathological analysis using hematoxylin and eosin (H&E) staining. Only samples with presence of distinct tumor tissue were used for further immunohistochemical analysis of protein expression. For immunohistochemical staining, the paraffin embedded samples were cut into 2 μm thin slices, applied on slides and dried for 30 min at 65°C in the incubator. After deparaffinization with xylene and a descending alcohol series, the tissue slides were pre-treated with 0.9% H_2_O_2_ in methanol to block endogenous peroxidases. Antigen demasking was proceeded via heat pre-treatment with Dako Target Retrieval Solution at either low (Dako K8005, Agilent, Santa Clara, CA, United States) or high (Dako K8002, Agilent, Santa Clara, CA, United States) pH depending on the antibody (low pH: ABCG2; high pH: FECH, CPOX, PPOX). Before incubation with the primary antibodies, tissue slides were treated with 10% fetal calve serum (Invitrogen 10108, Thermo Fisher Scientific, Waltham, MA, United States; 1:10 diluted in tris-buffered saline) to reduce unspecific binding sites. The primary antibodies (ABCG2 Abcam, Cambridge, United Kingdom; 1:500; FECH Proteintech, Manchester, United Kingdom; 1:350; PPOX Proteintech, Manchester, United Kingdom; 1:300; CPOX Proteintech, Manchester, United Kingdom; 1:500) were diluted (Antibody-Diluent DakoCytomation S2022, Agilent, Santa Clara, CA, United States) and incubated in a dark wet chamber at 4°C overnight. As secondary antibody detection kit, the Agilent EnVision™ HRP-Kit DakoCytomation K5007 was used. Counterstaining was performed by hematoxylin staining (#109249 Merck, Darmstadt, Germany). As control samples for the applied antibodies, we used tumor-free brain tissue for all antibodies as well as liver tissue for CPOX, kidney tissue for PPOX and heart tissue for FECH derived from autopsies. For assessment of protein expression of FECH, PPOX, CPOX, and ABCG2 all tissue slides were analyzed by two experienced neuropathologists (T.R., A.W.) and protein expression was semiquantitatively classified according to percentage of positive cells as low (0–20%), moderate (21–50%), or high (>50%) expression.

### mRNA Isolation, Transcription, and Quantitative Real Time PCR

In a further step, the second part of the obtained tissue samples stored at −80°C was utilized for mRNA analysis of the specific heme biosynthesis factors. For this purpose, isolation of mRNA was performed using ReliaPrep*™* RNA Tissue Miniprep System (Promega, Madison, WI, United States) according to the manufacturer’s protocol for non-fibrous tissue using frozen samples weighing 12–20 mg. Subsequently, spectroscopic analysis (Nanodrop 2000, Thermo Fisher Scientific, Waltham, MA, United States) of all mRNA isolates to assess mRNA concentration as well as 260/280 nm ratio was performed. Sample quality was considered sufficient if mRNA concentration was >60 ng/μl and 260/280 nm ratio was higher than 2.00. Reverse transcription into cDNA was conducted using RevertAid RT Reverse Transcription Kit (Thermo Fisher Scientific, Waltham, MA, United States) according to manufacturer protocol with 500 ng mRNA of each sample. PCR analysis was performed using Taqman Universal PCR Mastermix (Promega) as well as specific Taqman Probes for *CPOX* (Hs00164367_m1), *PPOX* (Hs00609392_m1), *ABCG2* (Hs01053790_m1), FECH (Hs01555261_m1), and β*2-microglobuline* (Hs00984230_m1; Thermo Fisher Scientific, Waltham, MA, United States). The PCR protocol included a 10-min initiation step at 95°C followed by 40 cycles of 30 s at 95°C (denaturation) and 60 s at 60°C. All samples and enzymes as well as non-template controls (5 μL of RNase free water added instead of a cDNA) were measured in triplicates. The mRNA expression results of the analyzed enzymes *FECH*, *CPOX*, *PPOX*, and *FECH* were calculated as log-fold change of delta cycle threshold (deltaCT) values using β2-Microglobuline as housekeeping gene.

### Statistical Analyses

Statistical analyses and figure preparation were conducted using IBM SPSS statistic software Version 26.0. Descriptive statistics included patient gender and age, fluorescence status, WHO tumor grade/subtype and IDH mutational status. The distribution of mRNA expression between fluorescing and non-fluorescing samples was analyzed using the unpaired *t*-test, while statistical testing of protein expression was performed using the Kendall-tau-b-test. In order to investigate the rate of successful mRNA to protein conversion, Kendall-tau-b coefficients of mRNA and protein expression levels were investigated. Statistical significance was assumed at the commonly applied threshold of *p* < 0.05.

## Results

Our study cohort for mRNA and protein expression analysis included altogether 40 tumor samples from surgery of 40 gliomas with either strong fluorescence or no visible fluorescence. In detail, 19 (47.5%) samples with strong 5-ALA induced fluorescence (*fluorescing group*) and 21 (52.5%) samples with no visible 5-ALA induced fluorescence (*non-fluorescing group*) were investigated. The *fluorescing group* consisted of 18 WHO grade IV gliomas (glioblastoma) and one WHO grade III glioma. The *non-fluorescing group* contained 17 WHO grade II gliomas and 4 WHO grade III gliomas. Detailed information on our study cohort is provided in Table 1.

**TABLE 1:** Patient characteristics.

	Overall		*Non-fluorescing*		*Fluorescing*	
	*n*	(%)	*n*	(%)	*n*	(%)
Number of patients	40	(100)	21	(52.5)	19	(47.5)
**Age**						
Median (range)	52 years (22–74)		40 years (22–59)		62 years (40–74)	
Gender (male: female)	1: 0.74		1: 0.5		1: 1.1	
**Localization**						
Frontal	18	(45.0)	11	(52.4)	7	(36.8)
Temporal	11	(27.5)	5	(23.8)	6	(31.6)
Parietal	6	(15.0)	1	(4.8)	5	(26.3)
Insular	2	(5.0)	2	(9.5)	−	−
Central	2	(5.0)	2	(9.5)	−	−
Occipital	1	(2.5)	−	−	1	(5.3)
**MRI contrast-enhancement**						
None	14	(35.0)	14	(66.6)	−	−
Patchy/faint	5	(12.5)	5	(23.8)	−	−
Focal	1	(2.5)	1	(4.8)	−	−
Nodular	1	(2.5)	1	(4.8)	−	−
Ring-like	19	(47.5)	−	−	19	(100)
**Preoperative PET**						
PET with hotspot	12	(30.0)	12	(57.1)	−	−
PET without hotspot	5	(12.5)	5	(23.8)	−	−
No PET performed	23	(57.5)	4	(19.1)	19	(94.7)
**Histopathological diagnosis**						
**WHO grade II**						
Astrocytoma IDH mut	7	(17.5)	7	(33.3)	−	−
Astrocytoma IDH wt	3	(7.5)	3	(14.3)	−	−
Oligodendroglioma IDH mut	7	(17.5)	7	(33.3)	−	−
** WHO grade III**						
Astrocytoma IDH mut	2	(5.0)	2	(9.5)	−	−
Astrocytoma IDH wt	2	(5.0)	1	(4.8)	1	(5.3)
Oligodendroglioma IDH mut	1	(2.5)	1	(4.8)	−	−
** WHO grade IV**						
Glioblastoma IDH mut	1	(2.5)	−	−	1	(5.3)
Glioblastoma IDH wt	17	(42.5)	−	−	17	(89.4)

*IDH, isocitratdehydrogenase; MRI, magnetic resonance imaging; mut, mutated; PET, positron emission tomography; WHO, World Health Organization; wt, wildtype.*

### mRNA Expression Analysis

**FIGURE 2:**
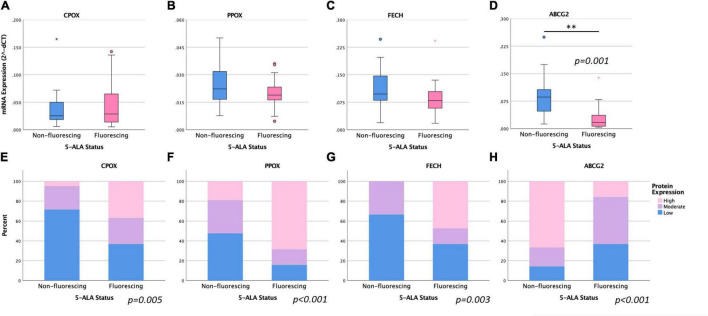
Comparison of mRNA and protein expression of intramitochondrial heme biosynthesis factors between tumor samples with visible 5-ALA fluorescence and absence of fluorescence. **(A–D)**: Boxplot diagrams are shown for the mRNA levels of CPOX **(A)**, PPOX **(B)**, FECH **(C)** and ABCG2 **(D)**. **(E–H)**: Bar Chart diagrams demonstrate the corresponding protein expression levels observed for CPOX **(E)**, PPOX **(F)**, FECH **(G)**, and ABCG2 **(H)**. For analyses that showed a significant difference between both groups, the respective *p*-values are included.

According to our mRNA expression analysis of specific enzymes of the heme biosynthesis, the resulting mean log-transformed deltaCT values were 0.059 ± 0.056 for *ABCG2*, 0.041 ± 0.038 for *CPOX*, 0.022 ± 0.010 for *PPOX* and 0.101 ± 0.055 for *FECH*. Regarding the visible 5-ALA induced fluorescence status, a significantly lower *ABCG2* mRNA expression was observed in the *fluorescing group* (0.029 ± 0.035) compared to the *non-fluorescing group* (0.086 ± 0.059; *p* = *0.001*). In contrast, no significant differences in the mRNA expression of *CPOX* (0.037 ± 0.035 vs. 0.045 ± 0.041, *p* = *0.512*), *PPOX* (0.025 ± 0.011 vs. 0.019 ± 0.009, *p* = *0.103*) and *FECH* (0.114 ± 0.057 vs. 0.086 ± 0.050, *p* = *0.107*) between the *non-fluorescing* and *fluorescing group* were present. Details are provided in Figures 2A–D and Table 2.

**TABLE 2:** mRNA and protein expression results.

	Overall		Non-fluorescing group		Fluorescing group		

**mRNA expression (2^∧^-dCT)**							
CPOX	0.041 ± 0.038		0.037 ± 0.035		0.045 ± 0.041		*p* = *0.512*
PPOX	0.022 ± 0.010		0.025 ± 0.010		0.019 ± 0.009		*p* = *0.103*
FECH	0.101 ± 0.055		0.114 ± 0.057		0.086 ± 0.050		*p* = *0.107*
ABCG2	0.059 ± 0.056		0.086 ± 0.059		0.029 ± 0.035		*p* = *0.001*

**Protein expression**	***n***	**(%)**	***n***	**(%)**	***n***	**(%)**	

CPOX							
Low	22	(100.0)	15	(68.2)	7	(31.8)	*p* = *0.005*
Moderate	10	(100.0)	5	(50.0)	5	(50.0)	
High	8	(100.0)	1	(12.5)	7	(87.5)	
PPOX							
Low	13	(100.0)	10	(77.0)	3	(23.0)	*p* < *0.001*
Moderate	10	(100.0)	7	(70.0)	3	(30.0)	
High	17	(100.0)	4	(23.5)	13	(76.5)	
FECH							
Low	21	(100.0)	14	(66.7)	7	(33.3)	*p* = *0.003*
Moderate	10	(100.0)	7	(70.0)	3	(30.0)	
High	9	(100.0)	0	(0.0)	9	(100.0)	
ABCG2							
Low	10	(100.0)	3	(30.0)	7	(70.0)	*p* = *0.001*
Moderate	13	(100.0)	4	(30.8)	9	(69.2)	
High	17	(100.0)	14	(82.4)	3	(17.6)	

### Protein Expression

#### Coproporphyrinogen Oxidase Protein Expression

Analysis of protein expression using immunohistochemical staining revealed a low CPOX protein expression in 22 samples of which 15 (68.2%) belonged to the *non-fluorescing group* and 7 (31.8%) belonged the *fluorescing group*. A high protein expression (>50%) of CPOX was present in 7 (88.5%) of 19 samples in the *fluorescing group* and in one (12.5%) of 21 specimens in the *non-fluorescing group*. Altogether, a significantly higher CPOX protein expression was found in the *fluorescing group* compared to the *non-fluorescing group* (*p* = 0.005; see Figure 2E and Table 2).

#### Protoporphyrinogen Oxidase Protein Expression

Low PPOX protein expression was observed in 10 (77.0%) samples in the *non-fluorescing group* and 3 (23.0%) specimens in the *fluorescing group*. A high protein expression of PPOX was present in 13 (76.5%) samples in the *fluorescing group* and in 4 (23.5%) specimens in the *non-fluorescing group*. Accordingly, we observed a significantly higher PPOX protein expression in the *fluorescing group* compared to the *non-fluorescing group* (*p* < 0.001; see Figure 2F and Table 2).

#### Ferrochelatase Protein Expression

Low protein expression of FECH was found in 14 (66.7%) samples in the *non-fluorescing group* and 7 (33.3%) specimens in the *fluorescing group.* A high protein expression of FECH was present in 9 (100.0%) samples in the *fluorescing group* and in none of the specimens in the *non-fluorescing group*. Altogether, a significantly higher FECH protein expression was observed in the *fluorescing group* compared to the *non-fluorescing group* (*p* = 0.003; see Figure 2G and Table 2).

#### ATP-Binding Cassette Subfamily B Member 2 Protein Expression

Low protein expression of ABCG2 was found in 3 (30.0%) samples in the *non-fluorescing group* and 7 (70%) specimens in the *fluorescing group.* High protein levels of ABCG2 were present in 14 (82.4%) samples in the non-*fluorescing group* and in 3 (17.6%) specimens in the *fluorescing group*. Overall, we detected a significantly lower ABCG2 protein expression in the *fluorescing group* compared to the *non-fluorescing group* (*p* < 0.001; see Figure 2H and Table 2).

### Correlation of mRNA and Protein Levels

**FIGURE 3:**
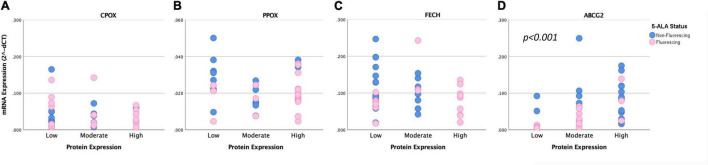
Correlation of mRNA and protein expression of intramitochondrial heme biosynthesis factors. Scatter plot diagrams show the correlation between mRNA and protein expression levels for CPOX **(A)**, PPOX **(B)**, FECH **(C)**, and ABCG2 **(D)**. For analyses that showed a significant difference between both groups, the respective p-values are included.

**FIGURE 4:**
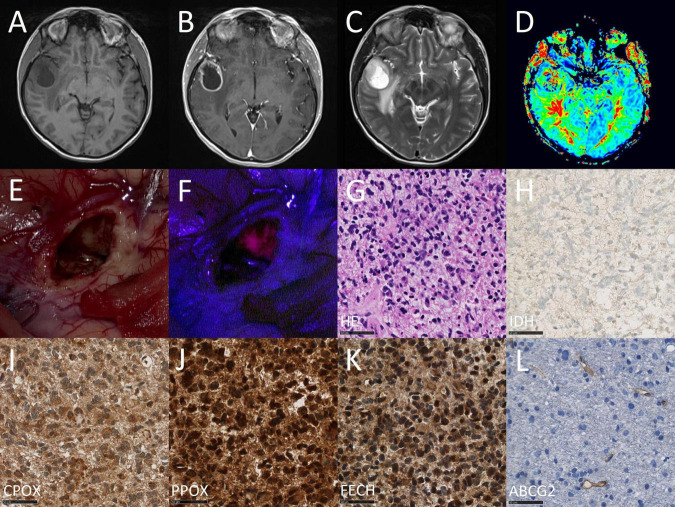
Case illustration of a typical patient from the fluorescing group. The images are obtained from the case of a 68-year-old female patient that underwent 5-ALA fluorescence-guided resection of a suspected high-grade glioma. In the first row, preoperative magnetic resonance imaging (MRI) including T1-weighted images before **(A)** and after **(B)** contrast-medium application as well as a T2-weighted image **(C)** and perfusion image **(D)** are shown. In the second row, the intraoperative situs is shown under conventional white light **(E)** and violet-blue excitation light **(F)** as well as routine histopathological analysis including H&E staining **(G)** and IDH mutation immunohistochemistry **(H)**. In the third row, study specific immunohistochemical stainings are shown including CPOX **(I)**, PPOX **(J)**, FECH **(K)**, and ABCG2 **(L)**.

**FIGURE 5:**
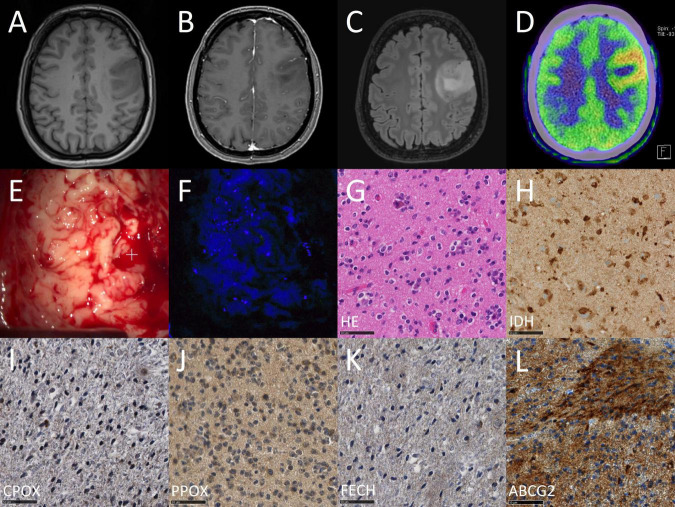
Case illustration of a typical patient from the non-fluorescing group. The images are obtained from the case of a 50-year-old female patient that underwent 5-ALA fluorescence-guided resection of a suspected low-grade glioma. In the first row, preoperative imaging including T1-weighted images before **(A)** and after **(B)** contrast-medium application as well as a T2-weighted image **(C)** and a ^11^C-methionine PET image **(D)** are shown. In the second row, the intraoperative situs is shown under conventional white light **(E)** and violet-blue excitation light **(F)** as well as routine histopathological analysis including H&E staining **(G)** and IDH mutation immunohistochemistry **(H)**. In the third row, study specific immunohistochemical stainings are shown including CPOX **(I)**, PPOX **(J)**, FECH **(K)**, and ABCG2 **(L)**.

Finally, we conducted a correlation between mRNA expression levels determined by quantitative PCR and protein expression assessed by immunohistochemistry of all analyzed enzymes. According to these data, we found a significant correlation between mRNA and protein expression for ABCG2 (tau: 0.485, *p* < 0.001). In contrast, no association was detected between mRNA and protein expression for the remaining enzymes including FECH (tau: −0.079, *p* = 0.539), PPOX (tau: −0.099, *p* = 0.757) and CPOX (tau: 0.046, *p* = 0.705). Scatter plots with correlation of mRNA and protein expression of enzymes are provided in Figure 3. Illustrative glioma cases with strong fluorescence and no fluorescence and corresponding protein expression analyses of intramitochondrial heme biosynthesis factors are shown in Figures 4, 5.

## Discussion

Nowadays, 5-ALA induced fluorescence is widely applied for intraoperative tumor visualization in the neurosurgical field (9, 10, 27). Initially, the presence of visible 5-ALA induced fluorescence was believed to be primarily determined by a disruption of the blood-brain barrier (BBB) (28, 29). Nevertheless, the occurrence of visible 5-ALA induced fluorescence was also observed in some pure LGG with intact BBB as evidenced by absent contrast-enhancement on preoperative MRI (15, 16, 30). The role of the BBB as sole predictor of 5-ALA induced fluorescence has therefore been questioned in the last years and additional influencing factors were thus investigated (31). Since visible fluorescence depends on metabolization of 5-ALA to fluorescent PpIX in gliomas, the heme biosynthesis pathway regulation seems to be a crucial influencing factor for fluorescence behavior (32). In this sense, prior investigations have suggested distinct effects of specific enzymes within the intramitochondrial heme metabolization such as CPOX and FECH on 5-ALA fluorescence behavior (21, 22). However, the exact alterations within the heme biosynthesis pathway resulting in visible 5-ALA induced fluorescence in different gliomas are still unclear.

### Present Study

We therefore designed the present study to evaluate promising enzymes of the intramitochondrial heme biosynthesis pathway to analyze their impact on visible 5-ALA induced fluorescence in different adult-type diffuse gliomas (WHO grades II-IV). For this purpose, we compared the mRNA as well as protein expression of CPOX, PPOX, FECH, and ABCG2 in 19 glioma tissue samples with presence of strong 5-ALA induced fluorescence during surgery to 21 non-fluorescing glioma samples. Since the aim of this study was to investigate the typical fluorescing and non-fluorescing gliomas undergoing 5-ALA guided procedures, no active selection for specific histopathological subtypes was performed. As expected from the fluorescence rates frequently reported in the literature (10, 14), glioblastomas thus accounted for a majority of fluorescing gliomas in our cohort, whereas most non-fluorescing gliomas were lower-graded astrocytomas.

### mRNA Expression

Regarding mRNA expression analysis, we found a significantly lower *ABCG2* mRNA expression in fluorescing samples compared to non-fluorescing samples. Similarly, Pustogarov et al. (21) reported lower values of *ABCG2* mRNA expression in fluorescing glioma cell cultures. While this difference did not reach statistical significance in the prior investigation of glioma cell lines, a statistically significant inverse correlation of ABCG2 expression levels with 5-ALA induced fluorescence has recently been demonstrated in gastrointestinal cancer cell lines (21, 33). In contrast, we observed no significant differences in mRNA expression levels of *CPOX*, *PPOX*, or *FECH* between fluorescing and non-fluorescing samples in our study. Interestingly, Pustogarov et al. (21) found a significantly lower *CPOX* mRNA expression in fluorescing glioma cells compared to non-fluorescing tumor cells. In contrary, Takahashi et al. (34) even described a significantly increased *CPOX* mRNA expression in fluorescing brain tumor samples. However, this study was finally retracted due to technical difficulties (35). In agreement with our present study, Pustogarov et al. (21) found no significant differences between fluorescing and non-fluorescing glioma specimens regarding *PPOX* and *FECH* mRNA expression. In a previous study, Teng et al. (22) reported a significantly lower *FECH* mRNA expression in glioblastoma tissue compared to LGG as well as normal brain tissue. In contrast, we recently found a significantly increased *FECH* mRNA expression in WHO grade IV compared to WHO grade II gliomas in a large “The Cancer Genome Atlas” (TCGA) cohort including 424 glioma samples (36). To our knowledge, our current analysis constitutes the first report describing a significantly lower *ABCG2* mRNA expression in fluorescing glioma samples derived from tumor resection.

### Protein Expression

Regarding protein expression, our study demonstrated a statistically significant increase in the protein expression of CPOX, PPOX, and FECH in fluorescing samples. In contrast, ABCG2 expression was significantly lower in fluorescing specimens. To our knowledge, no previous data comparing PPOX and ABCG2 protein expression in strongly fluorescing and non-fluorescing glioma specimens are available in the literature. With regard to CPOX protein expression, our observation of higher concentrations in fluorescing specimens is in line with an earlier investigation by Pustogarov et al. (21). Since glioblastoma typically show strong fluorescence, we surprisingly observed significantly higher rather than lower FECH protein expression in the fluorescing group. A possible explanation for these conflicting observations might be direct effects of preoperative 5-ALA administration on intratumoral FECH concentrations. However, this hypothesis needs to be further investigated in future studies analyzing the effects of preoperative 5-ALA administration on protein expression of heme biosynthesis factors.

### Association Between mRNA and Protein Expression

The results of this study demonstrate that protein levels of the investigated heme biosynthesis factors do not correlate with mRNA expression in gliomas in most cases (CPOX, PPOX, and FECH). While our findings constitute the first report on this interaction for PPOX and FECH in fluorescing and non-fluorescing glioma samples, our results for CPOX are well in accordance with earlier findings by Pustogarov et al. (21) reporting an decrease of *CPOX* on mRNA level, but increase of CPOX on protein level in fluorescing glioma cell lines. Solely ABCG2 showed a significant correlation of mRNA and protein levels in our study. While we can only speculate on the reason for the observed decoupling of mRNA and protein levels in CPOX, PPOX, and FECH, but not ABCG2, we consider it noteworthy that ABCG2 is the only investigated factor that is not specific to the heme biosynthesis pathway, but plays a relevant role in other metabolic processes as well (37–39).

### Effects of Protein Expression on 5-ALA Induced Fluorescence

**FIGURE 6:**
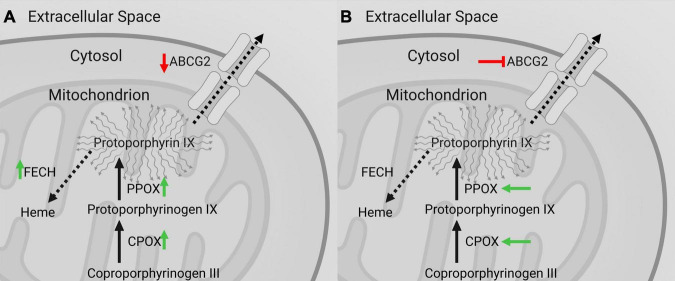
Observed effects on intramitochondrial heme biosynthesis factors in gliomas with visible 5-ALA fluorescence and promising pharmacological targets to optimize fluorescence visualization. **(A)** According to our study, heme biosynthesis pathway activity in general is enhanced in gliomas with visible 5-ALA fluorescence with upregulation of PpIX generating factors (CPOX and PPOX; *green arrow*) and decreased ABCG2 mediated PpIX efflux (*red arrow*) outweighing the also increased further metabolization of PpIX to heme by FECH (*green arrow*). **(B)** Intramitochondrial heme biosynthesis factors thus constitute promising pharmacological targets to optimize intraoperative 5-ALA fluorescence visualization of usually non-fluorescing tumor tissues such as low-grade gliomas. In this sense, enhancement of CPOX and PPOX (*green arrows*) as well as inhibition of ABCG2 (*red symbol*) represent promising candidates for future investigations. For example, the tyrosine kinase inhibitor lapatinib constitutes a potent suppressor of ABCG2 with an overall favorable safety profile in clinical use.

Most notably, the increases of CPOX and PPOX protein expression as well as decreases of ABCG2 protein expression in strongly fluorescing samples observed in our study should result in increased intracellular PpIX accumulation (40). In this sense, increased levels of CPOX and PPOX facilitate increased PpIX synthesis, whereas ABCG2 downregulation results in reduced PpIX efflux (see Figure 6A). More surprisingly, the observed increase of FECH protein levels in our study in fluorescing specimens promote the further metabolization of PpIX to FECH and thus its quenching that should theoretically result in lower rather than higher fluorescence intensity (22, 32). While, this observation seems paradoxical from a fluorescence standpoint, a complete upregulation of the heme biosynthesis pathway makes sense from a tumor biological point of view, since FECH rather than the intermediate metabolite PpIX is required for several cellular processes (41–43). Altogether, the results of this investigation show a general upregulation of heme biosynthesis pathway activity with increased enzyme expression and decreased PpIX efflux in fluorescing specimens on protein level. The intraoperatively observable fluorescence effect in gliomas seems thus to be a result of increased PpIX synthesis (PPOX, CPOX) and decreased efflux (ABCG2) outweighing its concurrently increased further metabolization to heme (FECH).

### Clinical Relevance and Future Directions

While 5-ALA induced fluorescence constitutes nowadays a routine tool for improved intraoperative detection of HGG tissue, other tumors such as LGG can usually not be visualized by this technique (11, 14). Identification of the underlying alterations resulting in intraoperatively detectable fluorescence is thus crucial, and modification of the fluorescence effect for example through silencing of specific heme biosynthesis factors has already been conducted in previous *in vitro* studies (22). In our present study, we were able to identify certain intramitochondrial heme biosynthesis factors as promising pharmacological targets to enhance the fluorescence effect. For this purpose, enhancement of CPOX and PPOX as well as inhibition of ABCG2 represent promising candidates for future investigations according to our current data (see Figure 6B). In our view, ABCG2 in particular constitutes a promising target, since suppressors in form of tyrosine kinase inhibitors such as lapatinib are already in clinical use and have an overall favorable safety profile (44, 45). Furthermore, potentially more specific inhibitors of ABCG2 activity like tariquidar are investigated in clinical trials (46). Future studies should thus focus on investigating the potential of specific pharmacological compounds to selectively improve the 5-ALA induced fluorescence effect in usually non-fluorescing tumor tissues such as LGG. Consequently, pharmacological modification of the heme biosynthesis pathway may ultimately extent the significance of 5-ALA induced fluorescence for tumor visualization in neurosurgery well beyond HGG.

### Limitations

The following limitations of this study should be kept in mind: (1) This investigation included 40 fluorescing and non-fluorescing gliomas of different histopathological grades and subtypes. The examined patient cohort reflects the commonly underlying glioma subtypes in fluorescing and non-fluorescing gliomas and a direct intratumoral comparison of fluorescence intensity is not easily possible due to the homogeneous fluorescence present in most gliomas. It needs to be kept in mind, however, that differences in tumor grade, subtype, and IDH status between the fluorescing and non-fluorescing subgroups were present in our cohort as well as they are in general glioma patient populations. Future investigations should thus strive to investigate the comparatively rare glioma cases where solid tumor areas with comparable histopathological characteristics exhibit different fluorescence qualities in order to investigate heme biosynthesis factor expression independently of tumor grade and subtype. (2) Moreover, we investigated a selection of four intramitochondrial heme biosynthesis factors deemed particularly likely to determine fluorescence behavior. While, our study successfully identified alterations in protein expression of four heme biosynthesis factors, the exact role of preceding metabolic steps and potentially even heme quenching constitutes a promising area for future research.

## Conclusion

In the present study, we investigated the mRNA as well as protein expression of promising intramitochondrial heme biosynthesis enzymes/transporters in glioma tissue samples of different fluorescence behavior. According to our data, we found distinct increases in the 5-ALA metabolizing enzymes CPOX, PPOX, and FECH and decreases in the PpIX exporting transporter ABCG2 on protein level in fluorescing glioma samples. Interestingly, only ABCG2 mRNA expression directly correlated with protein expression. Overall, these observations suggest a general upregulation of intramitochondrial heme biosynthesis activity in fluorescing glioma tissue. Intramitochondrial heme biosynthesis factors thus constitute promising pharmacological targets to optimize intraoperative fluorescence visualization and maximize safe resections especially in usually non-fluorescing tumors such as LGG.

## Data Availability Statement

The raw data supporting the conclusions of this article will be made available by the authors, without undue reservation.

## Ethics Statement

The studies involving human participants were reviewed and approved by Ethics committee of the Medical University Vienna. The patients/participants provided their written informed consent to participate in this study.

## Author Contributions

MM, DL-G, MM-M, AW, WB, GW, and BK contributed to conception and design of the study. MM and BK organized the database and performed the statistical analysis. MM, TR-P, NK, KB, RP, and AL performed histopahological and PCR analyses. JF and AB collected clinical data. MM wrote the first draft of the manuscript. NK, KB, BK, and GW wrote sections of the manuscript. All authors contributed to manuscript revision, read, and approved the submitted version.

## Conflict of Interest

AB had research support from Daiichi Sankyo and honoraria for lectures, consultation or advisory board participation from Roche Bristol-Meyers Squibb, Merck, Daiichi Sankyo as well as travel support from Roche, Amgen, and AbbVie. GW had received restricted travel support from NX Development Corp. The remaining authors declare that the research was conducted in the absence of any commercial or financial relationships that could be construed as a potential conflict of interest.

## Publisher’s Note

All claims expressed in this article are solely those of the authors and do not necessarily represent those of their affiliated organizations, or those of the publisher, the editors and the reviewers. Any product that may be evaluated in this article, or claim that may be made by its manufacturer, is not guaranteed or endorsed by the publisher.
